# Adaptive Studentized Multi-Kernel MMD for Cross-Condition Machinery Fault Diagnosis

**DOI:** 10.3390/s26217043

**Published:** 2026-11-07

**Authors:** Shiqian Wu, Weiming Zhang, Yurui Sang, Minghui Guo, Yuchen Lu, Yuxuan Zhang

**Affiliations:** 1 Shipbuilding Engineering Department, Jiangxi Polytechnic University, Jiujiang 332007, China; wsq_92@163.com (S.W.); 18270659009@163.com (W.Z.); yurui032@163.com (Y.S.); minghui_2007@126.com (M.G.); 2 Institute of Marine Equipment Structural Safety, Jiangxi Polytechnic University, Jiujiang 332007, China; 3 Yantai Research Institute, Harbin Engineering University, Yantai 264000, China; luyuchen@hrbeu.edu.cn; 4 College of Intelligent Science and Engineering, Beijing University of Agriculture, Beijing 102206, China; 5 Department of Computer and Electrical Engineering, Mid Sweden University, 85170 Sundsvall, Sweden

**Keywords:** fault diagnosis, unsupervised domain adaptation, Studentized maximum mean discrepancy, adaptive scheduling, domain-specific batch normalization, rotating machinery

## Abstract

This study proposes adaptive Studentized multi-kernel maximum mean discrepancy (MMD) for cross-condition machinery fault diagnosis. To account for sampling variation in mini-batch alignment, the multi-kernel MMD objective is normalized by empirical variability and integrated with domain-specific batch normalization. A label-free plateau criterion monitors fluctuations in the alignment loss and triggers joint cosine cooling of the learning rate and alignment weight, adapting the cooling onset to the observed training trajectory. The framework is evaluated on 18 window-level transfer tasks from the Paderborn bearing and PHM2009 gearbox datasets, with five random seeds per task and a fixed final-epoch reporting rule. Mean final-epoch accuracies reach 89.32% and 73.86%, exceeding DAN-style multi-kernel alignment by 2.16 and 2.39 percentage points, respectively. Compared with the fixed-schedule Studentized variant, adaptive scheduling improves accuracy by 0.57 and 0.37 percentage points, respectively. These results support combining variability-normalized alignment with label-free adaptive scheduling for cross-condition fault diagnosis.

## 1. Introduction

Vibration-based fault diagnosis is widely used to monitor the health condition of rotating machinery. In practical applications, however, the operating condition under which a diagnostic model is deployed is rarely identical to that used during model development. Variations in rotational speed, load, machine configuration, or transmission path can alter the statistical and spectral characteristics of vibration signals. As a result, a model trained under one operating condition may suffer a noticeable performance loss when applied to another condition. Collecting fault labels for every newly encountered operating condition is costly and often impractical [1,2,3,4,5]. These limitations have motivated the use of unsupervised domain adaptation (UDA), which transfers diagnostic knowledge from a labeled source domain to an unlabeled target domain [6,7,8,9].

A major line of UDA research reduces the discrepancy between source and target representations. The maximum mean discrepancy (MMD) provides a non-parametric measure of distribution difference in a reproducing kernel Hilbert space and has been incorporated into deep adaptation networks and joint adaptation networks [10,11,12]. Other methods use correlation alignment or adversarial learning to obtain representations that are less sensitive to the domain of origin [13,14,15]. These methods differ in the distributions they align and the objectives used to learn transferable representations.

The same development can be observed in machinery fault diagnosis. Early deep transfer methods introduced domain adaptation into diagnostic networks, while later studies employed multi-layer MMD, domain-specific information, feature disentanglement, or temporal–spectral representations to improve transfer across operating conditions [16,17,18,19]. These studies emphasize representation and alignment design. Training behavior also requires attention, because a higher final accuracy can coexist with substantial variation during optimization.

This issue is relevant because the MMD signal used during training is estimated from finite mini-batches. Its magnitude can therefore vary with the sampled data, the kernel scale, and the current feature distribution. At the same time, the classification loss and the alignment loss may provide different update directions as the model evolves. The resulting training process may exhibit noticeable variation in target-domain performance, particularly during the later epochs. The epoch associated with a high target-domain accuracy may also differ across transfer tasks and random seeds. When target labels are unavailable, target-domain performance cannot be used to decide which checkpoint should be retained. Selecting a model according to the highest target accuracy would introduce information that is not available in the intended unsupervised deployment setting. For this reason, a training protocol should distinguish between metrics used to report performance and signals used to control the optimization process. The importance of such protocol choices has also been emphasized in comparative studies of unsupervised transfer learning for machinery diagnosis [6].

Domain-specific batch normalization, multi-kernel MMD, and variance-normalized MMD address complementary parts of unsupervised adaptation. DSBN maintains domain-specific normalization statistics, multiple kernels capture discrepancies at different scales, and variance normalization scales the MMD statistic by an empirical variability estimate [11,20,21]. These components do not determine when to alter the learning rate or alignment weight. Here, that decision is based on recent variation in the unlabeled alignment loss.

The present study develops an adaptive statistical domain-adaptation framework for cross-condition fault diagnosis. Domain-specific batch normalization is used to absorb part of the low-order domain shift before feature alignment. The alignment objective is formulated as a Studentized multi-kernel MMD statistic, in which the estimated discrepancy is scaled by an empirical uncertainty term. For kernel selection, momentum-smoothed statistics set the kernel scales and weights, while current-batch statistics carry the alignment gradient. In addition, the learning rate and the alignment weight are adjusted according to the short-term variation of the unsupervised alignment loss. The adjustment is triggered only when the training signal satisfies a predefined plateau condition, rather than at a fixed epoch. Target-domain labels are excluded from optimization, scheduling, and checkpoint selection, and the raw model at the final epoch is used for the principal results.

The main contributions of this study are summarized as follows:

- We formulate a Studentized multi-kernel MMD objective for online domain alignment: the discrepancy is scaled by an empirical variability estimate, so the alignment signal accounts for the sampling variation of mini-batch estimates.
- Cooling begins when the recent variation of the unlabeled alignment loss falls below a preset threshold. The learning rate and alignment weight are then annealed together.
- The framework is evaluated on six directed PU transfers and 12 PHM2009 transfers with five random seeds per task. At the prespecified final epoch, it achieves mean accuracies of 89.32% and 73.86%, respectively, exceeding the fixed-schedule Studentized MK-MMD variant by 0.57 and 0.37 percentage points. The matched schedule ablation showed benchmark-dependent effects, with clearer improvement on PHM2009 than on PU.

The remainder of this paper is organized as follows. Section 2 reviews discrepancy-based domain adaptation, normalization-based adaptation, statistical two-sample testing, and related methods for machinery fault diagnosis. Section 3 presents the proposed framework, including domain-specific batch normalization, Studentized multi-kernel MMD, and label-free adaptive scheduling. Section 4 describes the datasets, transfer tasks, implementation details, and evaluation protocol. Section 5 reports the main comparison, ablation studies, and training-stage analysis. Section 6 interprets the component and scheduling findings and states the limitations of the present evaluation. Section 7 concludes the paper.

## 2. Related Work

### 2.1. Distribution Alignment in Deep Domain Adaptation

Deep domain adaptation methods differ in both the distributions they align and the way alignment is incorporated into representation learning. Early work brought MMD-based alignment into deep networks through Deep Domain Confusion [22]. Deep adaptation networks (DANs) apply multi-kernel maximum mean discrepancy (MK-MMD) to task-specific feature layers, while joint adaptation networks (JANs) extend this idea to joint feature–label distributions [11,12]. Other approaches measure domain discrepancy through second-order statistics, as in Deep CORAL, or learn domain-invariant representations through adversarial objectives, as in DANN and CDAN [13,14,15]. These methods provide complementary ways of controlling distribution shift; their choice of alignment objective is often tied to assumptions about which aspects of the source and target distributions should be shared.

### 2.2. Domain Adaptation for Machinery Fault Diagnosis

Domain adaptation has been studied extensively for machinery diagnosis under changes in operating conditions, machines, and fault severities. Reviews of this literature describe a range of transfer strategies, including feature-distribution alignment, adversarial learning, and methods that incorporate class or domain structure [6,8,23]. Early deep approaches established cross-domain diagnosis frameworks using labeled source data and unlabeled target data. For example, Lu et al. developed a deep model-based adaptation approach for fault diagnosis, while Zhang et al.’s domain-adaptive CNN (DACNN) explicitly minimized squared MMD between source and target representations during adaptation [24,25]. Guo et al. also investigated deep convolutional transfer learning for machine diagnosis when target-domain labels are unavailable [16].

Subsequent work explored richer alignment structures. Li et al. used multi-layer and multi-kernel MMD for rolling-bearing diagnosis, while Han et al.’s deep transfer network extended marginal alignment to joint distribution adaptation [17,26]. Other studies used class-related intermediate representations or higher-order feature statistics, including layered MMD and central-moment-discrepancy-based adaptation [27,28]. ADACNN combines adversarial learning with MMD in feature and predicted-label spaces [29]. More recent bearing-diagnosis methods incorporate domain-specific information or temporal–spectral representations [18,19]. In related industrial visual inspection, Han et al.’s SA-FPN uses scale-aware attention and feature refinement to improve small-defect detection on steel surfaces [30]. In electrical impedance tomography, DA-Net uses dense attention for lung image reconstruction [31], while DLEIP investigates unsupervised reconstruction using a deep-learning-based error image prior [32].

Recent studies have further developed cross-domain diagnosis through feature enhancement, reliability weighting, and progressive adaptation. Wang et al. combine attention-based feature enhancement and residual feature fusion with adversarial adaptation and MK-MMD for ball screw feed systems [33]. Ullah et al. use physics-guided sample reliability to weight representations and covariance alignment in cross-condition milling-machine diagnosis [34]. Qin et al. integrate progressive meta-learning, domain-adversarial learning, and MK-MMD regularization for multi-condition industrial processes [35].

Table 1 compares representative studies in terms of their alignment mechanisms, advantages, and design requirements. Alongside general UDA methods, it includes bearing-diagnosis approaches based on multi-layer matching, temporal–spectral learning, and hybrid adaptation.

The 2026 studies in Table 1 extend representation learning, sample weighting, or the adaptation procedure. The present method instead normalizes each mini-batch kernel discrepancy by its estimated variability and uses fluctuations in the unlabeled alignment loss to determine the onset of joint cooling. Sample reliability weights and empirical discrepancy-scale normalization therefore act on different quantities, while progressive adversarial adjustment and loss-triggered cooling provide different forms of training control.

### 2.3. Normalization, Statistical Alignment, and Weight Averaging

Normalization provides another way to account for domain differences. AdaBN recalculates batch-normalization statistics using target-domain data, whereas domain-specific batch normalization (DSBN) maintains separate normalization layers for different domains while retaining shared parts of the network [20,38]. In cross-condition diagnosis, domain-specific normalization can absorb part of the low-order feature shift, allowing subsequent representation learning and alignment to operate on the normalized features.

The statistical properties of MMD are also relevant to the choice of alignment objective. Sutherland et al. optimized a variance-normalized MMD criterion to improve the power of a two-sample test [21]. Building on this statistical perspective, the present study uses a Studentized multi-kernel MMD statistic as an online alignment objective.

Weight-averaging methods offer a related, but different, route to reducing variation during learning. Stochastic Weight Averaging (SWA) averages model parameters along an optimization trajectory, and Mean Teacher uses an exponential moving average of the student weights to form consistency targets [39,40]. SWAD applies dense, overfit-aware weight averaging in domain generalization [41]. These methods operate primarily in parameter space or through teacher–student consistency. By contrast, the scheduling mechanism considered here uses the variation of an unlabeled alignment-loss signal to determine when to reduce the learning rate and alignment weight.

### 2.4. Training Schedules and Unsupervised Model Selection

Training schedules also determine how adaptation objectives change during optimization. DANN ramps the adversarial coefficient according to normalized training progress, whereas DDA adjusts the relative weights of marginal and conditional discrepancies [14,42].

A separate line of work addresses model selection when target labels are unavailable. Deep Embedded Validation (DEV) estimates target risk using adapted representations; MixVal uses mixed target samples as probes; and EnsV studies ensemble-based selection for unlabeled target domains [43,44,45]. These methods rank candidate models or hyperparameter settings. The controller studied here instead adjusts the training schedule from an unlabeled signal, while the reported endpoint is fixed in advance.

## 3. Methodology

**Figure 1:**
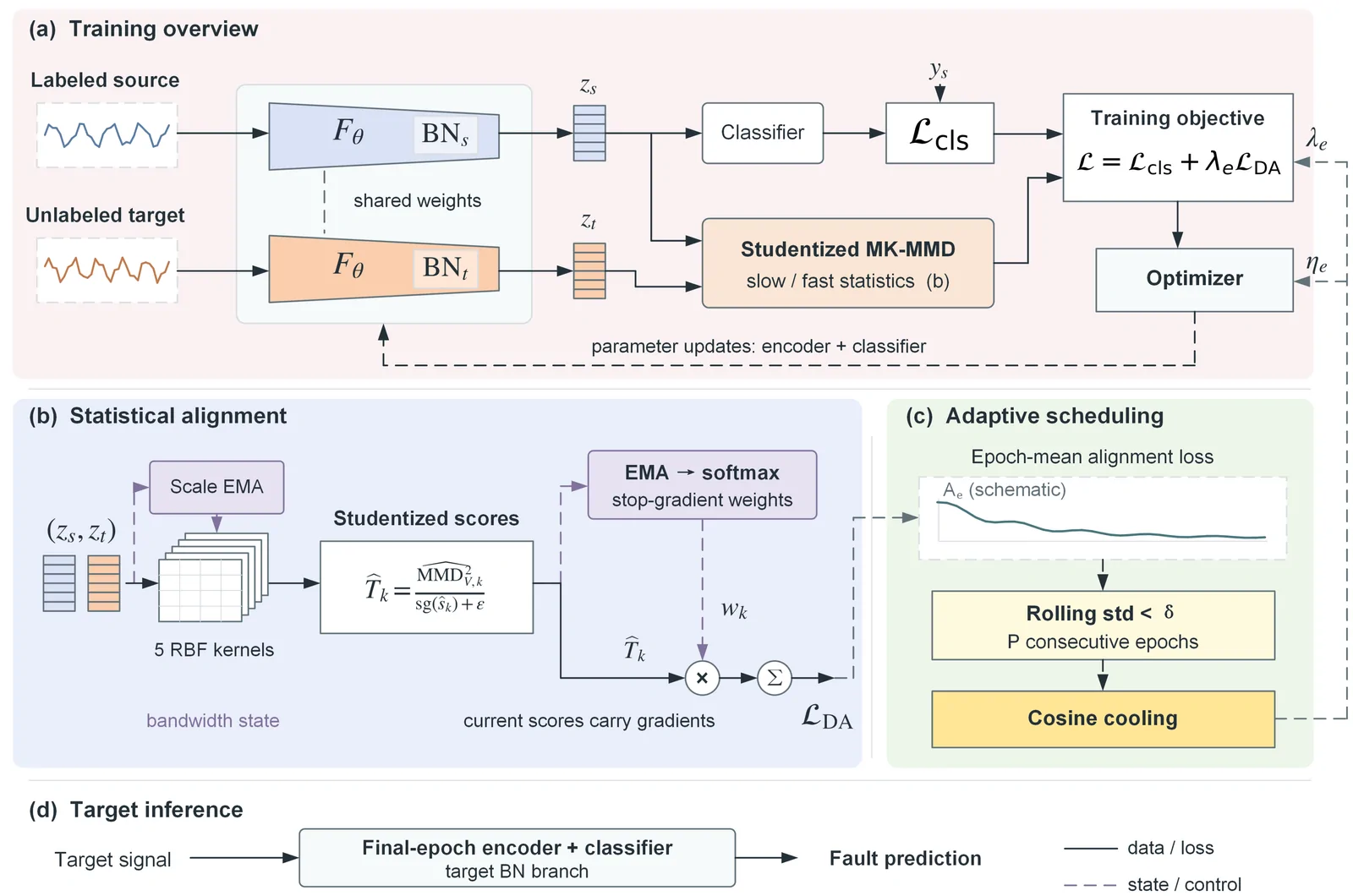
Overview of the proposed training and inference workflow. (**a**) Source-supervised warm-up with target-branch normalization updates, followed by unlabeled adaptation. (**b**) Studentized multi-kernel MMD with current-batch gradients and smoothed kernel statistics. (**c**) Unlabeled-loss-variation-triggered cooling of the learning rate and alignment weight. (**d**) Inference with the unaveraged final-epoch model. Signal traces and the alignment-loss curve are schematic.

The procedure begins with signal windowing and data partitioning, followed by channel-wise standardization fitted on the source training subset. Source-supervised warm-up first trains the encoder and classifier, while unlabeled target batches update the target BN statistics. Adaptation then combines source classification with Studentized multi-kernel alignment of source and target features. During adaptation, a plateau criterion on the recent variation of the unlabeled alignment loss triggers joint cosine cooling of the learning rate and alignment weight. Training continues to the prescribed final epoch, whose unaveraged model is used for target inference with the target BN branch. Figure 1 summarizes these stages and their data and control paths. The following subsections specify the network architecture, alignment objective, kernel-statistic updates, scheduling, and model reporting.

The main methodological developments are shown in Figure 1b,c. Panel (b) constructs the online alignment objective by normalizing each kernel discrepancy by its estimated variability and combining the resulting Studentized scores; panel (c) uses the recent variation of the unlabeled alignment loss to determine when joint cosine cooling of the learning rate and alignment weight begins. The alignment loss thus links the statistical objective to the scheduling decision within the training framework in panel (a).

### 3.1. Problem Setting and Network Architecture

We consider closed-set unsupervised domain adaptation for fault diagnosis. The source domain contains labeled vibration segments $D_{s}={\left\{\left(x_{i}^{s},y_{i}^{s}\right)\right\}}_{i=1}^{N_{s}}$, whereas the target adaptation set contains unlabeled segments $D_{t}={\left\{x_{j}^{t}\right\}}_{j=1}^{N_{t}}$. The two domains share the same fault categories but correspond to different operating conditions. The objective is to learn a classifier using source labels and unlabeled target signals, without using target labels for parameter updates or training-schedule decisions. For each input channel, the mean and standard deviation are estimated from the source training set and then applied to both domains.

The network consists of a shared one-dimensional convolutional feature extractor, a feature projection layer, and a linear classifier. The feature extractor contains a convolutional stem and four depthwise-separable residual blocks. Global average pooling is followed by a linear projection, layer normalization, and a GELU activation. Let $f_{\theta }\left(x;d\right)$ denote the resulting feature vector for domain d∈{s,t}, and let $g_{\phi }$ denote the classifier. Source and target samples pass through the same convolutional and classifier parameters, but use separate batch-normalization branches. This domain-specific batch normalization (DSBN) structure follows Chang et al. [20]; the pseudo-label training procedure used in their original framework is not adopted here.

For a source mini-batch $B_{s}$, the supervised classification loss is

(1) $$L_{\mathrm{cls}}=-\frac{1}{|B_{s}|}\sum_{\left(x_{i}^{s},y_{i}^{s}\right)\in B_{s}}\log p_{\theta ,\phi }\left(y_{i}^{s}|x_{i}^{s};s\right).$$

Here, $p_{\theta ,\phi }\left(y_{i}^{s}|x_{i}^{s};s\right)$ is the softmax probability assigned to the true class $y_{i}^{s}$ for input $x_{i}^{s}$, with *s* indicating the source BN branch and $|B_{s}|$ denoting the source mini-batch size. Minimizing the mean negative log-probability trains the feature extractor and classifier to distinguish the source-domain fault categories.

During the source-supervised warm-up, unlabeled target samples are still forwarded through the target branch so that its batch-normalization statistics are updated. They do not contribute an alignment gradient until the adaptation stage begins.

The warm-up establishes source-supervised features before discrepancy optimization. Its duration is examined in Section 5.

The source-derived channel-wise standardization remains a fixed input transformation for both domains throughout training and inference. DSBN then normalizes intermediate features, with the target branch accumulating running statistics from unlabeled target adaptation batches, including during warm-up. At target inference, the final-epoch model uses the target BN branch in evaluation mode, so its stored running statistics are used without updates from target-test batches.

### 3.2. Studentized Multi-Kernel Alignment

Maximum mean discrepancy (MMD) measures the difference between distribution mean embeddings in a reproducing kernel Hilbert space [10]. We compute an MMD estimate at several Gaussian-kernel scales, following the use of multiple kernels in deep domain adaptation [11]. For each adaptation step, the source and target feature mini-batches are truncated to the same size $n=\min(n_{s},n_{t})$. We write their features as ${\left\{z_{i}^{s}\right\}}_{i=1}^{n}$ and ${\left\{z_{i}^{t}\right\}}_{i=1}^{n}$.

The kernel bandwidth is determined from the positive pairwise squared distances in the pooled source–target feature batch. Let $a_{b}$ be their median at adaptation step *b*. To reduce batch-to-batch variation in this scale, we maintain

(2) $${\bar{a}}_{b}=\beta_{a}{\bar{a}}_{b-1}+\left(1-\beta_{a}\right)a_{b},\;{\bar{a}}_{1}=a_{1},$$

where $\beta_{a}$ is a momentum coefficient. For kernel *k*, with a fixed scale multiplier $m_{k}$, we use

(3) $$\kappa_{k,b}\left(u,v\right)=\exp\left(-\frac{{∥u-v∥}_{2}^{2}}{2m_{k}{\bar{a}}_{b}}\right).$$

The bandwidth scale ${\bar{a}}_{b}$ is detached from the computational graph.

For each kernel, the squared discrepancy is computed with the biased mini-batch MMD estimator, including the diagonal terms in the within-domain kernel matrices:

(4) $$\begin{array}{cc} {\hat{D}}_{k,b}= & \frac{1}{n^{2}}\sum_{i=1}^{n}\sum_{j=1}^{n}\kappa_{k,b}\left(z_{i}^{s},z_{j}^{s}\right)+\frac{1}{n^{2}}\sum_{i=1}^{n}\sum_{j=1}^{n}\kappa_{k,b}\left(z_{i}^{t},z_{j}^{t}\right)\\ \; & -\frac{2}{n^{2}}\sum_{i=1}^{n}\sum_{j=1}^{n}\kappa_{k,b}\left(z_{i}^{s},z_{j}^{t}\right). \end{array}$$

A nonnegative clamp is applied in the implementation to avoid small negative values caused by numerical error.

Variance-normalized MMD criteria have been investigated for improving the power of kernel two-sample tests [21]. Motivated by this statistical principle, we normalize the current discrepancy using an empirical scale estimate. Specifically, the per-sample kernel contribution is

(5) $$\begin{array}{cc} h_{i,k,b}= & \frac{1}{n}\sum_{j=1}^{n}\kappa_{k,b}\left(z_{i}^{s},z_{j}^{s}\right)+\frac{1}{n}\sum_{j=1}^{n}\kappa_{k,b}\left(z_{i}^{t},z_{j}^{t}\right)\\ \; & -\frac{1}{n}\sum_{j=1}^{n}\kappa_{k,b}\left(z_{i}^{s},z_{j}^{t}\right)-\frac{1}{n}\sum_{j=1}^{n}\kappa_{k,b}\left(z_{j}^{s},z_{i}^{t}\right). \end{array}$$

Its mean equals the unclamped estimate in Equation (4). With  ${\bar{h}}_{k,b}=n^{-1}\sum_{i}h_{i,k,b}$, we define   

(6) $${\hat{v}}_{k,b}=\frac{1}{n}\sum_{i=1}^{n}{\left(h_{i,k,b}-{\bar{h}}_{k,b}\right)}^{2},\;{\hat{s}}_{k,b}=\sqrt{\frac{{\hat{v}}_{k,b}}{n}+\epsilon },$$

and obtain the normalized discrepancy

(7) $$T_{k,b}=\frac{{\hat{D}}_{k,b}}{{\hat{s}}_{k,b}+\epsilon }.$$

Here ϵ>0 is a numerical constant. The variance and scale terms in Equation (6) are detached during backpropagation. Because the per-sample contributions share observations within a mini-batch, ${\hat{s}}_{k,b}$ is treated as an empirical normalization term rather than an exact standard-error estimate. Accordingly, $T_{k,b}$ is optimized as an alignment objective and is not interpreted as a calibrated two-sample test statistic.

### 3.3. Slow Statistical State and Current-Batch Gradient

A kernel weight determined solely by the current mini-batch can vary substantially between optimization steps. We therefore maintain a slowly updated statistic for each kernel:

(8) $${\bar{T}}_{k,b}=\beta_{T}{\bar{T}}_{k,b-1}+\left(1-\beta_{T}\right)\mathrm{stopgrad}\left(T_{k,b}\right),\;{\bar{T}}_{k,1}=\mathrm{stopgrad}\left(T_{k,1}\right).$$

The kernel weights are calculated from this statistical state,

(9) $$w_{k,b}=\mathrm{stopgrad}\left[\frac{\exp({\bar{T}}_{k,b}/\tau )}{\sum_{\ell =1}^{K}\exp\left({\bar{T}}_{\ell ,b}/\tau \right)}\right],$$

where *K* is the number of kernels and τ controls the concentration of their weights. A kernel with a larger normalized discrepancy receives a larger weight.

The alignment loss, however, uses the discrepancies from the current mini-batch:

(10) $$L_{\mathrm{DA},b}=\sum_{k=1}^{K}w_{k,b}T_{k,b}.$$

Thus, the slow state determines kernel weighting, whereas the current features provide the optimization gradient. Since the bandwidth, empirical scale estimates, and kernel weights are detached, they are not themselves optimized through Equation (10). This separation limits changes in kernel selection caused by individual mini-batches without replacing the current alignment signal with an averaged loss.

### 3.4. Adaptive Scheduling from an Unlabeled Training Signal

At epoch *e*, the training objective for each mini-batch is

(11) $$L_{e,b}=L_{\mathrm{cls},b}+\lambda_{e}L_{\mathrm{DA},b},$$

where $\lambda_{e}$ controls the contribution of domain alignment. For the first *W* epochs, $\lambda_{e}=0$ and the network is trained with source labels. During the subsequent *R* epochs, $\lambda_{e}$ rises smoothly from zero to $\lambda_{\max}$:

(12) $$\lambda_{e}=\frac{\lambda_{\max}}{2}\left[1-\cos\left(\pi \frac{e-W}{R}\right)\right],\;W<e\leq W+R.$$

Unless a cooling event has been triggered, $\lambda_{e}$ remains at $\lambda_{\max}$ after this ramp. The learning rate is $\eta_{\mathrm{src}}$ during warm-up and $\eta_{\mathrm{adapt}}$ thereafter.

The cooling event is determined only from the observed, unweighted alignment loss. Let $B_{e}$ be the set of mini-batches at epoch *e*, and let $n_{b}^{s}$ be the source-batch size. We compute the epoch-level signal

(13) $$A_{e}=\frac{\sum_{b\in B_{e}}n_{b}^{s}\;L_{\mathrm{DA},b}}{\sum_{b\in B_{e}}n_{b}^{s}}.$$

Only adaptation epochs are included in this signal. In all experiments, the rolling window contains H=5 adaptation epochs. Once *H* such values are available, their rolling standard deviation is

(14) $$S_{e}={\left[\frac{1}{H}\sum_{j=e-H+1}^{e}{\left(A_{j}-{\bar{A}}_{e}\right)}^{2}\right]}^{1/2},\;{\bar{A}}_{e}=\frac{1}{H}\sum_{j=e-H+1}^{e}A_{j}.$$

When $S_{e}<\delta$ for *P* consecutive eligible epochs, cooling begins at the next epoch, denoted by $e_{★}$.

After the trigger, both the learning rate and alignment weight follow a cosine-shaped cooling profile. Cosine annealing is a conventional learning-rate schedule [46]; here, the start of the cooling phase is determined by the unlabeled training signal rather than fixed in advance. For $e\geq e_{★}$, define

(15) $$q_{e}=\frac{e-e_{★}+1}{E-e_{★}+1},\;c_{e}=\frac{1+\cos(\pi q_{e})}{2},$$

where *E* is the predetermined final epoch. The two scheduled quantities are

(16) $$\eta_{e}=\eta_{\min}+\left(\eta_{\mathrm{adapt}}-\eta_{\min}\right)c_{e},\;\lambda_{e}=\lambda_{\mathrm{final}}+\left(\lambda_{\max}-\lambda_{\mathrm{final}}\right)c_{e}.$$

If the plateau criterion is never satisfied, cooling is not triggered and the pre-trigger settings are retained. Hence, the trigger epoch adapts to the observed training signal, while the total training duration and post-trigger cosine profile remain predefined.

### 3.5. Optimization and Model Reporting

The network is optimized with AdamW as implemented in PyTorch 2.5.1 [47], and the gradient norm is clipped at 5. The numerical settings for training duration, kernel scales, momentum statistics, and plateau detection are summarized in Table 2.

Target-domain labels are reserved for retrospective evaluation and are not used in optimization, scheduling, or checkpoint selection. The reported model is the unaveraged network at the prespecified final epoch.

## 4. Experimental Setup

### 4.1. Datasets and Transfer Tasks

Experiments were conducted on two public machinery-diagnosis benchmarks. The first is the Paderborn University (PU) bearing dataset, which provides measurements under different bearing conditions and operating settings [48]. We selected 13 bearing states: K001, K002, K003, KA04, KA15, KA16, KA22, KA30, KI04, KI14, KI16, KI18, and KI21. Three operating conditions were considered: N15_M07_F10, N09_M07_F10, and N15_M01_F10, corresponding to different combinations of rotational speed and load. The input contains one vibration channel and two motor-current channels. Each acquisition record is approximately 4 s long. We used measurement run 1 for each selected bearing state and operating condition, giving 13 records per domain. All six directed source-to-target transfers among the three conditions were evaluated.

The second benchmark is the labeled expanded release of the PHM Society 2009 gearbox dataset [49]. We used the input-side acceleration signal and eight diagnostic classes. The experiments were restricted to 30 Hz and considered two load levels with two measurement runs per load. These four domains formed 12 directed transfer tasks. Because the shaft speed was fixed, this protocol evaluates shifts associated with load and measurement runs rather than cross-speed generalization.

Table 3 compares the characteristics of PU, PHM2009, and CWRU. PU and PHM2009 were selected to evaluate the same alignment and scheduling framework on bearing and gearbox diagnosis, respectively, using different signal-channel configurations and domain definitions. The PU transfers vary rotational speed or load, while the PHM2009 transfers vary load and measurement run at fixed speed, providing complementary settings for examining the behavior of the statistical alignment objective and training schedule.

Class labels identify the recorded mechanical state. In PU, each of the 13 bearing-state identifiers listed above defines a separate class, with integer labels 0–12 following that order. Thus, the three healthy reference bearings also retain separate state identifiers. In PHM2009, labels 0–7 correspond to the supplied spur 1–spur 8 configurations in numerical order. Each configuration describes the joint state of the gearbox components and can include multiple simultaneous faults. These class assignments are inherited from the bearing-state records and the labeled gearbox release [48,49]. Classification accuracy in Equation (17) measures agreement with these reference labels.

The class label *y* and domain index *d* describe different attributes. The label specifies the mechanical state to be diagnosed, while the domain index identifies the acquisition setting: rotational speed and load for the selected PU domains, and load and measurement run at 30 Hz for PHM2009. A class retains the same label across domains, allowing its signals to change with operating condition while the diagnostic task remains fixed. Table 4 lists the class and domain encodings used in the experiments.

**Figure 2:**
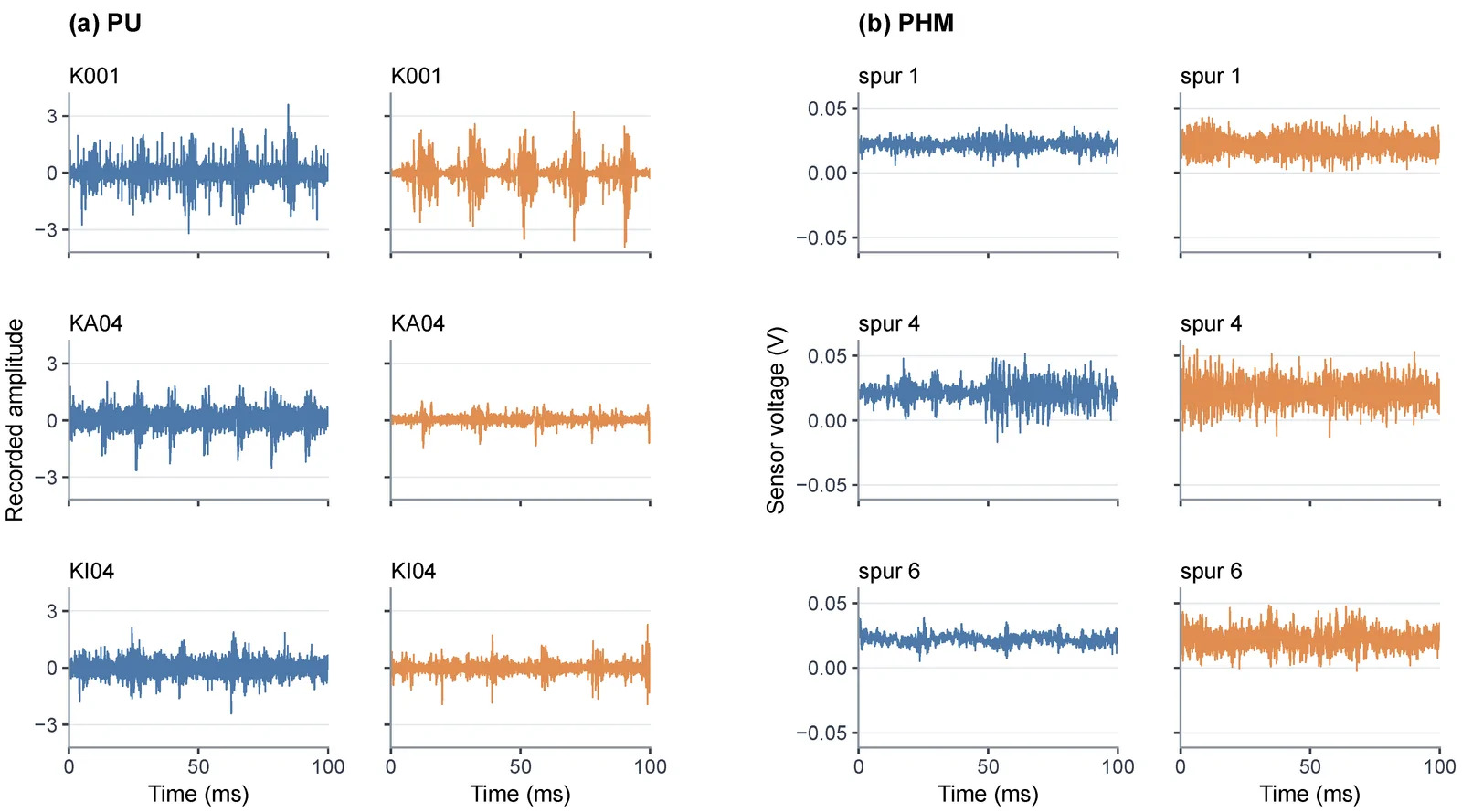
Recorded signal examples: (**a**) PU bearing states and (**b**) PHM2009 gearbox configurations. Within each panel, the left and right columns show PU recordings at 1500 and 900 rpm, respectively, or PHM2009 recordings at high and low load at 30 Hz. State identifiers appear above the traces. The same 100 ms interval, beginning 0.5 s into measurement run 1, is shown without filtering or amplitude rescaling. Amplitude limits are shared within each dataset. PU uses the recorded time coordinates and stored vibration amplitudes; PHM2009 shows input-side sensor voltage with time calculated from the documented sampling rate.

Figure 2 shows recorded traces for three classes from each dataset under two acquisition settings. The PU examples use the vibration channel of K001, KA04, and KI04 at 1500 and 900 rpm; the PHM2009 examples use input-side acceleration for spur 1, spur 4, and spur 6 at high and low load. Changes in amplitude and temporal structure are visible both between states and across acquisition settings for the same state, illustrating why class identity and operating condition are represented separately.

For the PU dataset, each signal was segmented into non-overlapping windows of 3072 samples, yielding 1079, 1079, and 1090 windows in the three selected domains, respectively. For PHM2009, non-overlapping windows of 1024 samples were used, yielding 2080 windows per domain. For every task and random seed, windows in each domain were randomly divided into an 80% training/adaptation subset and a 20% held-out subset. The source subset was divided into labeled training and source-test data; the target subset was divided into unlabeled adaptation data and target-test data. Channel-wise normalization statistics were calculated from the source training subset and applied unchanged to the other subsets.

The partitioning unit was a segmented window. Different subsets could therefore contain non-overlapping windows from the same acquisition record and retain characteristics specific to that recording. This protocol evaluates window-level transfer between the selected operating-condition or acquisition domains, with held-out windows drawn from their respective acquisition pools. All methods were compared using identical partitions for each task and seed. Generalization to independently acquired runs requires evaluation with complete records held out.

### 4.2. Comparison Methods and Implementation Details

The evaluation compares the proposed adaptive Studentized MK-MMD method with a source-only baseline and five UDA comparators: DAN-style MK-MMD [11], DAN + cosine, CDAN [15], Minimum Class Confusion (MCC) [36], and fixed-schedule Studentized MK-MMD. All UDA methods use the same one-dimensional convolutional backbone and data partitions; the source-only model uses shared batch normalization and does not process target adaptation batches.

Each method was evaluated on the six PU and 12 PHM2009 transfers using five random seeds (42–46), yielding 30 and 60 runs per method, respectively. Every run lasted 50 epochs with a batch size of 128, a backbone width of 32, and a 64-dimensional projected feature. AdamW with a weight decay of $10^{-4}$ was used throughout, and the gradient norm was clipped at 5. For the UDA methods, the first 20 epochs comprised source-supervised warm-up at a learning rate of $10^{-3}$; adaptation began at $5\times 10^{-4}$. Method-specific settings are given in Table 2.

The adaptation weights in Table 2 multiply different objectives and are therefore not directly comparable by numerical value.

Computational environment. Experiments were conducted on a Windows 10 Enterprise workstation equipped with an Intel Core i5-12600KF CPU, 64 GB RAM, and an NVIDIA GeForce RTX 4060 GPU with 8 GB VRAM. The software environment comprised Python 3.11.15, PyTorch 2.5.1, and CUDA 12.1.

All methods were trained for the fixed 50-epoch budget, and the unaveraged model at epoch 50 was used for the reported accuracy and macro-F1. No checkpoint was selected or restored according to target-test performance. Target-test labels were used to record per-epoch diagnostic accuracy and to calculate the retrospective stability measures below, including the peak-to-final gap. These quantities did not enter parameter updates, adaptive scheduling, or checkpoint selection; the plateau controller used only the unlabeled alignment-loss trajectory.

### 4.3. Evaluation Metrics and Statistical Analysis

Classification performance was measured using accuracy and macro-averaged F1-score. For *N* target-test samples, accuracy is

(17) $$\mathrm{Accuracy}=\frac{1}{N}\sum_{i=1}^{N}I\left({\hat{y}}_{i}=y_{i}\right),$$

where $y_{i}$ and ${\hat{y}}_{i}$ denote the true and predicted labels, respectively. For class *c*, the F1-score is

(18) $$F1_{c}=\frac{2\;{\mathrm{TP}}_{c}}{2\;{\mathrm{TP}}_{c}+{\mathrm{FP}}_{c}+{\mathrm{FN}}_{c}},\;\mathrm{Macro}-F1=\frac{1}{C}\sum_{c=1}^{C}F1_{c},$$

where *C* is the number of classes. For each metric *M*, the mean over seeds was first calculated within each transfer task, and the task means were then averaged with equal weight:

(19) $$\bar{M}=\frac{1}{T}\sum_{t=1}^{T}\left(\frac{1}{S}\sum_{s=1}^{S}M_{t,s}\right),$$

where *T* and S=5 denote the number of transfer tasks and random seeds.

Seed-to-seed variability was calculated within each task and then averaged across tasks:

(20) $$\begin{array}{cc} {\bar{M}}_{t} & =\frac{1}{S}\sum_{s=1}^{S}M_{t,s},\\ {\mathrm{SD}}_{\mathrm{seed}}\left(M\right) & =\frac{1}{T}\sum_{t=1}^{T}\sqrt{\frac{1}{S-1}\sum_{s=1}^{S}{\left(M_{t,s}-{\bar{M}}_{t}\right)}^{2}}. \end{array}$$

Means are reported as percentages and seed SDs as percentage points.

Training stability was summarized using two retrospective measures. Let $a_{t,s,e}$ be the target-test accuracy for task *t*, seed *s*, and epoch *e*. The within-run standard deviation over the final ten epochs is

(21) $$\sigma_{t,s}^{\mathrm{last}10}=\sqrt{\frac{1}{10}\sum_{e=41}^{50}{\left(a_{t,s,e}-{\bar{a}}_{t,s}^{\mathrm{last}10}\right)}^{2}},$$

where ${\bar{a}}_{t,s}^{\mathrm{last}10}$ is the mean accuracy over epochs 41–50. The peak-to-final gap is

(22) $$G_{t,s}=\max_{1\leq e\leq 50}a_{t,s,e}-a_{t,s,50}.$$

Both measures are calculated from target-test diagnostics and averaged over task–seed runs. They describe variation within a training run, whereas Equation (20) describes variation across repeated runs.

For paired method comparisons, let $\Delta_{t,s}=M_{t,s}^{\left(A\right)}-M_{t,s}^{\left(B\right)}$ denote the difference between two methods evaluated on the same task and seed. We computed 95% paired seed-bootstrap confidence intervals for the equal-weight mean performance difference across the fixed transfer-task set. In each of 10,000 replicates, we resampled five paired seed differences with replacement within every task, calculated their task-wise means, and averaged those means equally across tasks. The interval bounds were the 2.5th and 97.5th percentiles. The task set was held fixed throughout, so the intervals quantify uncertainty in the mean difference due to seed variability conditional on the evaluated tasks.

## 5. Results

### 5.1. Diagnostic Performance Across Transfer Tasks

Table 5 and Table 6 report final-epoch performance separately for the two datasets. On PU, the adaptive method achieved 89.32% accuracy and 88.89% macro-F1. Its accuracy was 0.57 percentage points higher than that of fixed-schedule Studentized MK-MMD (95% paired seed-bootstrap CI for the six PU tasks: 0.21–0.94), with a task-averaged seed SD of 5.33 percentage points compared with 5.27 for the fixed-schedule variant. The accuracy gains over DAN-style alignment and DAN + cosine were 2.16 and 5.20 percentage points.

On PHM2009, the adaptive method reached 73.86% accuracy and 74.12% macro-F1. Its accuracy gain over fixed-schedule Studentized MK-MMD was 0.37 percentage points (95% paired seed-bootstrap CI: 0.25–0.50), and the corresponding seed SDs were 3.28 and 3.18 percentage points. The gains over DAN-style alignment and DAN + cosine were 2.39 and 2.69 percentage points. The paired intervals describe seed variability for the evaluated transfer tasks.

**Figure 3:**
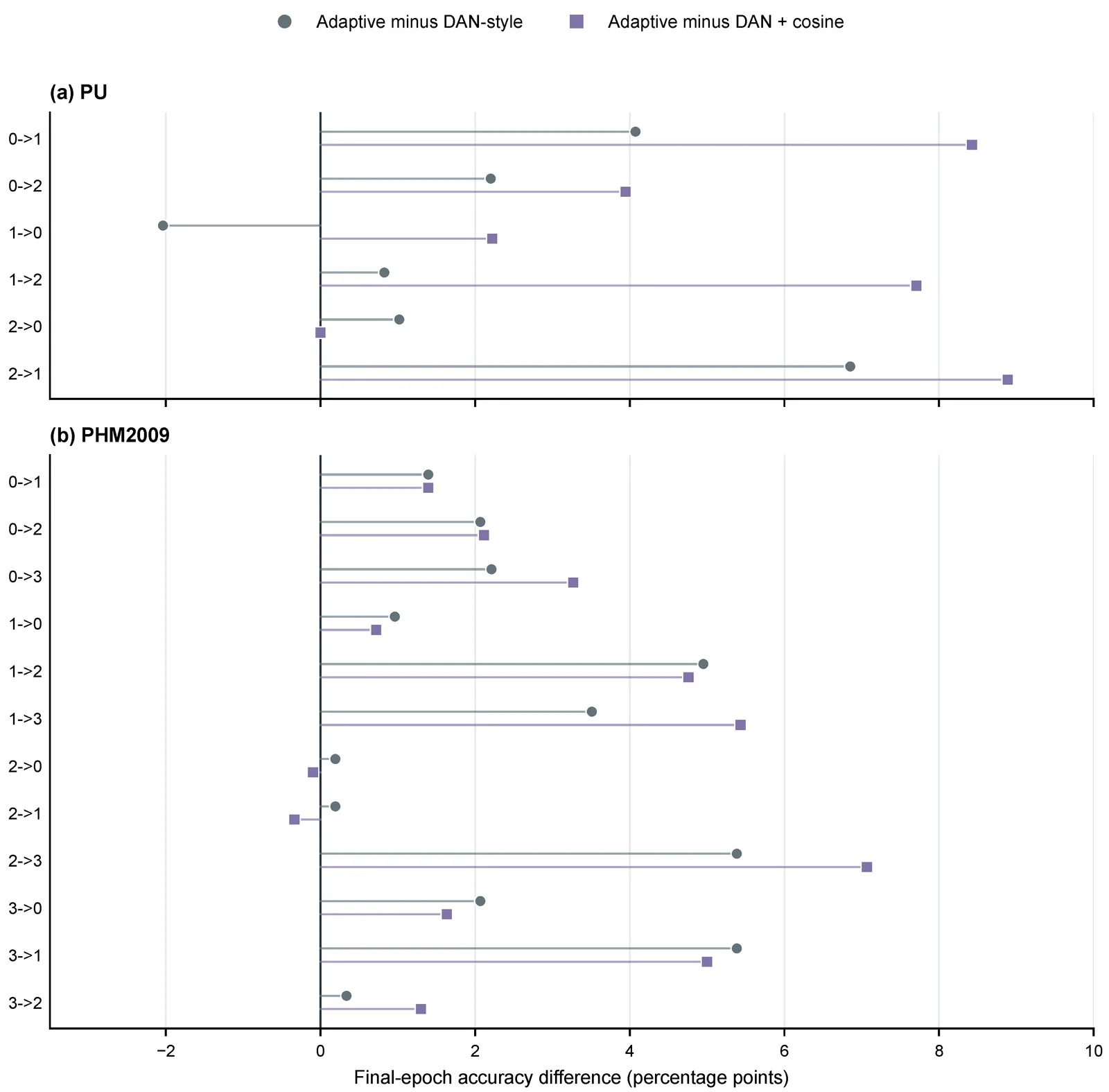
Task-wise differences in final-epoch target-test accuracy between the adaptive method and two DAN-based comparators. Each point is the difference between five-seed mean accuracies for one directed transfer task; positive values favor the adaptive method. The numbers on the vertical axes denote operating-condition or acquisition-domain indices defined in Section 4.

The accuracy gains over fixed-schedule Studentized MK-MMD were accompanied by macro-F1 gains of 0.71 percentage points on PU and 0.41 on PHM2009. Thus, the endpoint improvements appear in both sample-level accuracy and class-averaged F1. Figure 3 further resolves the comparisons with the DAN-based methods by transfer direction. The adaptive method exceeded DAN-style alignment in five of six PU tasks and all 12 PHM2009 tasks. Against DAN + cosine, it performed better in five PU tasks and tied in one; on PHM2009, it performed better in ten tasks and worse in two. These task-wise results show that the mean gains over the DAN-based comparators were supported by improvements across multiple transfer directions.

### 5.2. Training Dynamics and Final-Epoch Reporting

The last-10-epoch measures in Table 5 and Table 6 describe variation within individual runs. On PU, the adaptive method had a last-10-epoch SD of 0.94 percentage points, compared with 0.29 for the fixed-schedule variant and 0.27 for DAN + cosine. The corresponding values on PHM2009 were 0.59, 0.33, and 0.24 percentage points. The adaptive method therefore had higher late-stage variation than these scheduled comparators. Its peak-to-final gap was 1.77 percentage points on PU and 1.17 on PHM2009.

**Figure 4:**
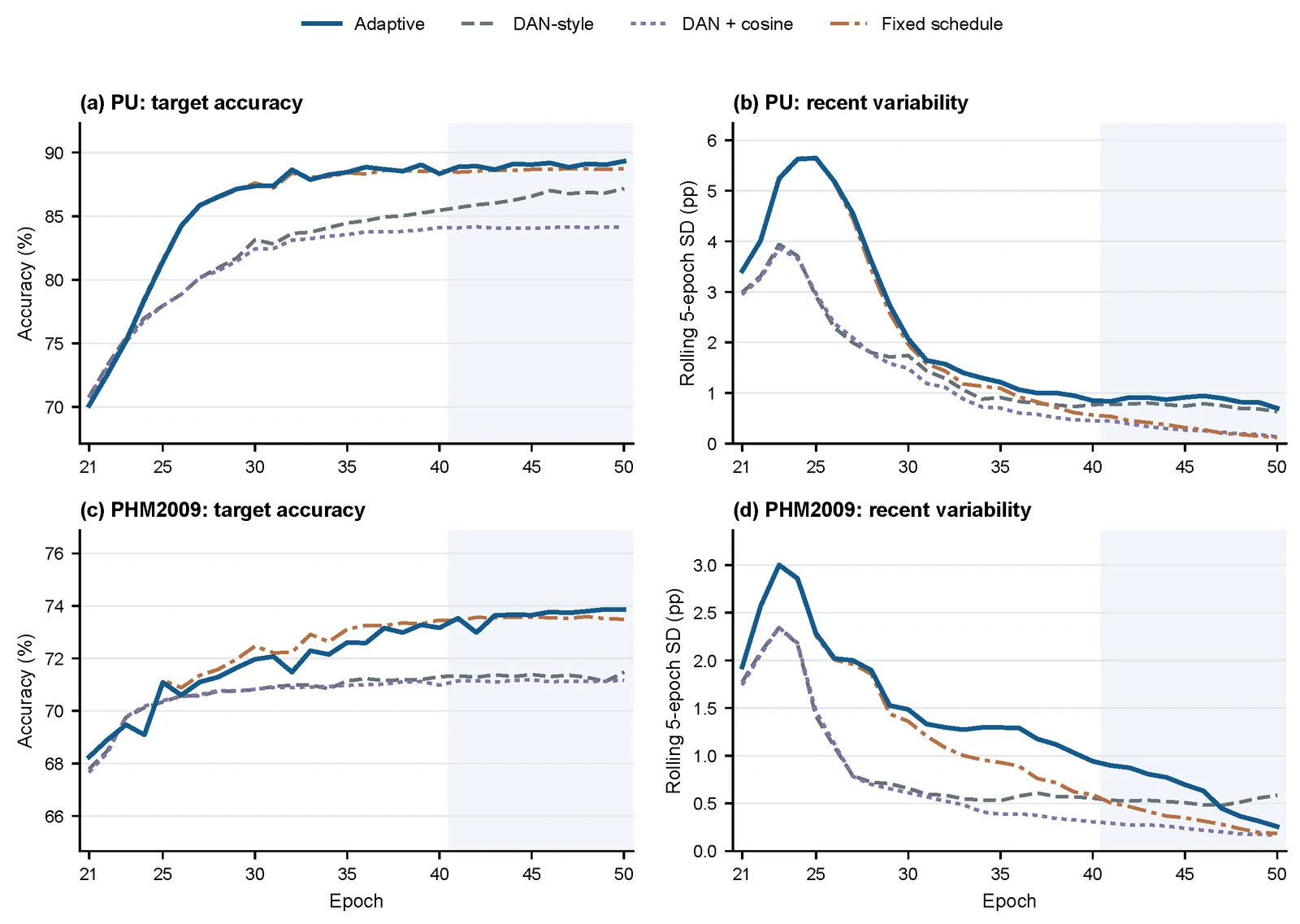
Training-stage target-test accuracy and recent performance variability on PU and PHM2009. Each curve is averaged across transfer tasks and seeds. Recent variability is the mean of within-run rolling five-epoch accuracy SDs, distinct from the seed SD and last-10-epoch SD reported in Table 5 and Table 6. The shaded region denotes epochs 41–50.

Figure 4 connects these endpoint results to the trajectories over adaptation epochs 21–50. For fixed-schedule Studentized MK-MMD, cooling follows a predetermined timetable; the adaptive method retains its pre-cooling settings until the unlabeled loss criterion is met. Runs therefore experience different durations of cooling. Cosine-cooled DAN and fixed-schedule Studentized MK-MMD produced smoother late-stage trajectories, while the adaptive method reached higher mean final accuracy, particularly on PU. The comparison shows that final accuracy and temporal variation respond differently to the training schedule. Section 5.3 examines the cooling controls separately, and Section 6 relates their effects to the recorded trigger times.

### 5.3. Component and Scheduling Analysis

The component comparisons use the same input segmentation and standardization, convolutional layer configuration, and task–seed data partitions. The component ablations use fixed-schedule Studentized MK-MMD as their reference, while the targeted training-control analyses compare settings on matched task–seed subsets. Final diagnostic performance is considered alongside variation across repeated seeds, temporal variation within runs, and changes in the kernel-selection state to characterize the effects of these choices.

Table 7 and Table 8 compare the statistical-alignment components and scheduling configurations. On PU, removing domain-specific batch normalization reduced final accuracy from 88.75% to 35.96%, while removing Studentization reduced it to 78.19%. On PHM2009, the corresponding accuracies were 69.39% and 69.92%, compared with 73.49% for the fixed-schedule reference. Removing slow kernel-selection statistics changed mean accuracy by less than 0.1 percentage points on either dataset. Domain-specific normalization had the largest effect on PU, while Studentization improved final accuracy on both benchmarks within this reference configuration. The effect of slow statistics is more evident in the kernel-weight trajectories examined below.

In the full-task schedule comparison in Table 7, the no-cooling configuration reached 89.65% accuracy on PU, compared with 89.32% for the proposed schedule; its last-10-epoch SD was also higher (1.55 vs. 0.94 percentage points). We then compared joint cooling, learning-rate-only cooling, alignment-weight-only cooling, and no cooling on the same 0→1 and 0→2 transfers with the same three seeds and pre-cooling settings. Table 9 reports these matched subset results; their absolute means differ from the full-task averages in Table 7 and Table 8. Relative to matched no cooling, joint cooling changed PU accuracy and last-10-epoch SD by +0.23 and +0.07 percentage points, respectively; on PHM2009, the corresponding changes were +0.96 and −0.82 percentage points.

On PHM2009, learning-rate-only cooling reduced last-10-epoch SD from 1.35 to 0.56 percentage points, close to the 0.53 points obtained with joint cooling; alignment-weight-only cooling yielded 1.01 points. The three cooling configurations achieved similar final accuracies (79.49–79.61%), compared with 78.65% without cooling. On PU, learning-rate-only cooling had the highest final accuracy (91.53%), while the last-10-epoch SDs of the three cooling configurations were similar (0.98–1.02 points).

We varied the plateau threshold and patience around the settings used in the main experiments, changing one parameter at a time while retaining the five-epoch rolling window. The same two transfers and three seeds per transfer were used for each setting. Table 10 reports final accuracy, late-stage variation, and cooling duration.

On PU, reducing δ to 0.10 prevented cooling in these six runs, whereas increasing it to 0.20 yielded 11.0 cooling epochs on average. Across the five settings, final accuracy ranged from 90.84% to 91.38% on PU and from 79.41% to 79.65% on PHM2009. The threshold had a clearer effect on cooling duration than on final accuracy in this targeted analysis; the patience settings produced smaller changes in cooling duration.

We also varied the source-supervised warm-up at 0, 10, and 20 epochs while keeping the total training duration at 50 epochs. Table 11 shows that the 20-epoch setting attained the highest final target accuracy and the lowest final-epoch alignment loss on both benchmarks. The same ordering held for target accuracy on each of the four evaluated transfers.

**Figure 5:**
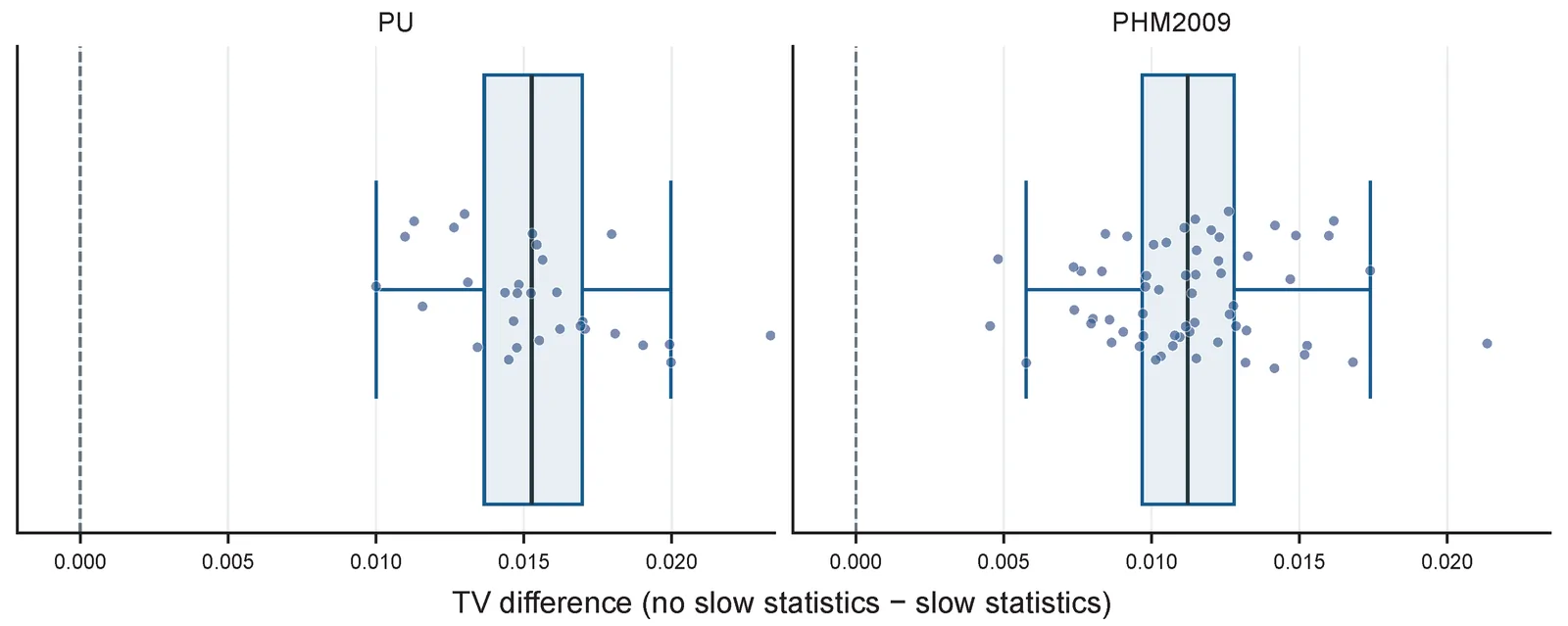
Paired task–seed differences in kernel-weight variation for fixed-schedule Studentized MK-MMD. Each point represents one matched run (30 PU and 60 PHM2009 pairs). Variation is the mean half-L1 distance between consecutive epoch-averaged kernel-weight vectors over epochs 21–50; positive values indicate lower variation with slow statistics. The box spans the interquartile range, and the central line marks the median.

Figure 5 compares paired kernel-weight variation under the fixed schedule. The plotted difference is computed within each matched task–seed pair as the mean kernel-weight variation without slow statistics minus that with slow statistics; positive values therefore indicate reduced variation with slow statistics. Slow statistics reduced mean variation from 0.02714 to 0.01172 on PU and from 0.02096 to 0.00964 on PHM2009, relative reductions of 56.8% and 54.0%, respectively.

The no-cooling configuration had greater late-stage variation than the fixed-schedule configuration on both datasets. Its final accuracy was higher on PU (89.65% vs. 88.75%) but lower on PHM2009 (72.68% vs. 73.49%). Since the learning rate and alignment weight changed together, this comparison evaluates their joint schedule.

The unlabeled plateau criterion produced different activation patterns across the two benchmarks. A trigger was recorded in 16 of 30 adaptive PU runs, including two triggers at the final epoch; thus, 14 runs entered cooling before training ended. On PHM2009, all 60 runs triggered before the final epoch, with a mean trigger epoch of 35.0. These records describe when the prescribed controller acted.

### 5.4. Effect of Available Record Duration

To examine the influence of available data, we compared the full-record setting with a setting that retained source training and target adaptation windows only when their end points lay within the first half of each acquisition record, corresponding to approximately 2 s of available recording per record. Input windows remained 3072 samples long. For each task and seed, source-test and target-test window indices were identical in the two settings, and normalization statistics were fitted on the retained source training subset. Both settings used the adaptive method with the same network and 50-epoch training configuration on PU transfers 0→1 and 0→2, with three seeds per transfer.

Table 12 shows that restricting the available record duration approximately halved the training and adaptation window counts. Mean accuracy decreased by 3.84 percentage points, while macro-F1 decreased from 90.90% to 87.02%. Accuracy decreased in all six matched runs. The half-record setting retained 87.46% accuracy at the same input-window length.

## 6. Discussion

The component ablations show how domain-specific normalization and statistical scaling of the alignment objective affect final accuracy. Removing DSBN reduced final accuracy by 52.79 percentage points on PU and 4.10 points on PHM2009 relative to fixed-schedule Studentized MK-MMD. DSBN maintains separate normalization branches for the source and target domains, allowing each to accumulate its own feature moments. The pronounced PU reduction is consistent with the importance of this domain-specific normalization in the selected transfers. Removing Studentization reduced accuracy by 10.56 and 3.57 points, respectively. Studentization scales each kernel discrepancy by its estimated variability; the ablation supports including this statistical scaling in the alignment objective on both benchmarks.

Slow statistics smooth the state used to select kernel scales and weights, while current-batch scores carry gradients. Their removal changed final accuracy by less than 0.1 percentage points on either dataset, whereas retaining them reduced mean epoch-to-epoch kernel-weight variation by 56.8% on PU and 54.0% on PHM2009. Their principal observed effect was smoother kernel selection.

On the full PU task set, the no-cooling configuration had 0.33 percentage points higher final accuracy than the proposed schedule (89.65% vs. 89.32%), while the proposed schedule had 0.61 percentage points lower last-10-epoch SD (0.94 vs. 1.55). On the matched PU subset, joint cooling changed final accuracy by +0.23 points and last-10-epoch SD by +0.07 points relative to no cooling. On PHM2009, the corresponding changes were +0.96 and −0.82 points. The controller used the same rolling window (five epochs), threshold (δ=0.15), and patience (three consecutive epochs) across tasks, with cooling triggered by the unlabeled alignment-loss trajectory.

The trigger records help interpret the different scheduling outcomes. With the shared controller settings, 14 of 30 PU runs entered cooling before training ended, whereas all 60 PHM2009 runs did so, with a mean trigger epoch of 35.0. PHM2009 therefore typically received a substantial cooling phase within the 50-epoch budget; many PU runs retained the pre-cooling settings throughout training or reached the trigger only at the endpoint. The same controller consequently applied different amounts of cooling across the two benchmarks. The matched schedule comparisons in Table 9 then distinguish the effects of the two controls within the evaluated subsets.

The one-factor cooling comparisons indicate that learning-rate cooling accounts for most of the late-stage variation reduction on PHM2009, while alignment-weight cooling has a smaller effect.

For deployment without target labels, δ and *P* can be set from the behavior of the unlabeled alignment-loss signal. With the loss definition and five-epoch rolling window fixed, practitioners can inspect $S_{e}$ on unlabeled development runs, place δ in a sustained low-variation range, and use *P* to exclude isolated dips while leaving enough epochs for cooling. The selected values are then kept fixed across transfer tasks. This calibrates the controller’s timing from an observable training signal without using target-domain accuracy to select parameters.

The source-only comparison reflects the complete adaptation protocol. The source-only model uses shared batch normalization and does not process target adaptation batches, whereas the UDA methods use DSBN and unlabeled target data. Its contrast with the adapted models therefore includes both target-specific normalization and distribution alignment. CDAN’s low mean accuracy was accompanied by substantial variation across seeds and within runs, particularly on PU, where its seed SD was 21.17 percentage points and its last-10 SD was 8.56 points. Its low mean accuracy was therefore accompanied by inconsistent performance across seeds and epochs.

The evaluation used window-level splits after segmentation, so windows from one acquisition record could occur in different subsets. Record-level splits are needed to assess transfer to independent recordings.

## 7. Conclusions

An adaptive Studentized multi-kernel MMD framework was developed for cross-condition machinery fault diagnosis. Domain-specific normalization was combined with an alignment objective in which each kernel discrepancy was scaled by its estimated variability. Joint cosine cooling of the learning rate and alignment weight was triggered by recent fluctuations in the unlabeled alignment loss. The unaveraged model at the prespecified final epoch was used for reporting, without target labels being used for optimization, scheduling, or checkpoint selection.

Mean final-epoch accuracies of 89.32% on PU and 73.86% on PHM2009 were obtained across 18 directed transfers with five seeds per task. Gains of 2.16 and 2.39 percentage points over DAN-style alignment, and 0.57 and 0.37 points over fixed-schedule Studentized MK-MMD, were recorded, respectively. The roles of domain-specific normalization and Studentization were supported by the component ablations, while slow statistics were associated primarily with smoother kernel selection. In the matched schedule analysis, a clearer benefit from joint cooling was observed on PHM2009, with higher accuracy and lower late-stage variation; smaller, mixed changes were observed on PU.

The effects of plateau settings, warm-up duration, and available record duration were further characterized through controlled experiments. The combination of variability-normalized alignment and label-free adaptive scheduling was supported by the evaluated window-level transfers. Future validation will be extended to independent acquisition records and additional machinery benchmarks.

## Figures and Tables

**Table 1:** Representative domain-adaptation studies: mechanisms, advantages, and limitations or design requirements.

Study	Main Mechanism	Advantages	Limitations or Design Requirements
DANLong et al. (2015) [11]	Multi-kernel MMD matching at task-specific layers.	Captures discrepancies at multiple kernel scales within feature learning.	Mean-embedding matching does not explicitly impose class-conditional alignment.
CDANLong et al. (2018) [15]	Adversarial adaptation conditioned on features and classifier predictions.	Uses class-related information to address multimodal distributions; entropy conditioning accounts for prediction uncertainty.	Requires a domain discriminator and incorporates classifier predictions into the alignment process.
MCCJin et al. (2020) [36]	Minimization of pairwise class confusion in target predictions.	Needs no domain discriminator and can complement other adaptation objectives.	Confusion estimates are constructed from target prediction probabilities, with temperature scaling and uncertainty weighting.
Multi-layer DALi et al. (2019) [17]	Multi-kernel MMD alignment at multiple layers of a bearing-diagnosis CNN.	Constrains source–target discrepancies at several representation levels.	Requires selecting the aligned layers and combining their discrepancy terms with source classification.
TSDANDing et al. (2024) [19]	Temporal and spectral branches with Sinkhorn-divergence-based adaptation.	Learns and aligns complementary temporal and spectral representations.	Uses a dedicated spectral neural network alongside the temporal branch.
DS-HDA NetJiao et al. (2025) [37]	Time–frequency feature fusion, marginal and conditional adaptation, and soft pseudo-labeling.	Combines complementary signal information with global and class-related adaptation.	Requires frequency-feature construction, soft pseudo-label generation, and joint configuration of several loss terms.
FE-MK-DANNWang et al. (2026) [33]	Attention and residual feature fusion with adversarial adaptation and MK-MMD.	Combines feature enhancement with explicit and adversarial distribution matching.	Requires feature-enhancement modules and a domain discriminator; demonstrated on ball screw systems.
Trust-aware DAUllah et al. (2026) [34]	Physics-guided sample reliability weighting and covariance alignment.	Accounts for differences in signal reliability during representation learning and alignment.	Requires physics-guided descriptors and a learned reliability estimator.
MDAPMLQin et al. (2026) [35]	Progressive meta-learning, adversarial adaptation, and MK-MMD regularization.	Combines cross-task learning with explicit distribution alignment.	Requires meta-learning and adversarial training; evaluated on multi-condition industrial processes.

**Table 2:** Adaptation objectives and method-specific training settings.

Method	Adaptation Objective	Method-Specific Schedule and Settings
Source-only	None	Source classification only; shared batch normalization; learning rate $10^{-3}$ for all 50 epochs.
DAN-style	Multi-kernel MMD	Five Gaussian-kernel scale multipliers {0.25,0.5,1,2,4}; the alignment weight is linearly ramped to 1.0 over five epochs and then held fixed.
DAN + cosine	Multi-kernel MMD	Uses the same Gaussian-kernel scale multipliers as DAN-style. After the 20-epoch source warm-up, the learning rate follows a cosine schedule from $5\times 10^{-4}$ to $10^{-5}$; the alignment weight is ramped to 1.0 over five epochs and then cosine-decayed to 0.4.
CDAN	Conditional adversarial loss	Multilinear feature–prediction conditioning; a domain discriminator with 128 hidden units; no entropy conditioning; adaptation weight increased linearly to 1.0 over five epochs.
MCC	Target class-confusion loss	Softmax temperature of 2.5; adaptation weight increased linearly to 1.0 over five epochs.
Fixed-schedule Studentized MK-MMD	Studentized multi-kernel MMD	Uses DSBN and slow/fast kernel statistics. After the 20-epoch source warm-up, the learning rate follows a cosine schedule from $5\times 10^{-4}$ to $10^{-5}$; the MMD weight is ramped to $\lambda_{\max}=0.05$ over five epochs and then cosine-decayed to $\lambda_{\mathrm{final}}=0.02$.
Proposed method	Studentized multi-kernel MMD	Uses the same alignment components as the fixed-schedule variant. Cooling begins when the five-epoch rolling standard deviation of the unlabeled alignment loss remains below 0.15 for three consecutive epochs; the learning rate and MMD weight then follow cosine schedules. The kernel-statistic momentum parameters are $\beta_{a}=0.90$ and $\beta_{T}=0.95$, with τ=1.

**Table 3:** Characteristics of PU, PHM2009, and CWRU and their use in this study. Fault settings, signals, and operating conditions describe the available datasets; the final column specifies the experimental subsets used here.

Dataset	Machinery and Fault Settings	Available Signals	Operating Conditions	Use in This Study
PU [48]	Rolling bearings: healthy states, artificial damage, and damage from accelerated lifetime tests.	Synchronized vibration and motor-current signals, with supporting operating measurements.	Four settings defined by rotational speed, load torque, and radial force.	Thirteen bearing states and three conditions; one vibration and two current channels; six directed transfers.
PHM2009 [49]	Gearbox test rig with healthy and faulty component configurations.	Input- and output-side acceleration and a tachometer signal.	Five shaft speeds (30–50 Hz), two load levels, and repeated runs.	Eight diagnostic classes at 30 Hz; two loads and two runs per load; input-side acceleration; 12 directed transfers.
CWRU [50]	Motor bearings: healthy states and single-point defects introduced by electro-discharge machining.	Acceleration measured at drive-end and fan-end locations; base measurements in some experiments.	Motor loads of 0–3 hp, with corresponding changes in rotational speed.	Dataset-level comparison.

**Table 4:** Class-label and domain-index encodings used in the experiments. PU torque and radial force are given in N m and N, respectively; all PHM2009 domains use 30 Hz shaft speed.

Dataset	Class Label *y*: State Identifier	Domain Index *d*: Acquisition Setting
PU	0: K001; 1: K002; 2: K003; 3: KA04; 4: KA15; 5: KA16; 6: KA22; 7: KA30; 8: KI04; 9: KI14; 10: KI16; 11: KI18; 12: KI21.	0: 1500 rpm, 0.7 N m, 1000 N; 1: 900 rpm, 0.7 N m, 1000 N; 2: 1500 rpm, 0.1 N m, 1000 N.
PHM2009	Labels 0–7 correspond to spur 1–spur 8, respectively.	0: high load, run 1; 1: high load, run 2; 2: low load, run 1; 3: low load, run 2.

**Table 5:** Final-epoch performance and temporal measures on PU. Accuracy and macro-F1 are reported as the mean ± task-averaged sample SD across five seeds (mean in %, SD in percentage points). Last-10 SD and peak–final gap describe within-run variation in percentage points. Bold mean values indicate the highest mean accuracy and macro-F1 in the table.

Method	Accuracy (Mean ± SD)	Macro-F1 (Mean ± SD)	Last-10 SD (pp)	Peak–Final Gap (pp)
Source-only	16.22 ± 4.25	8.32 ± 2.46	0.37	10.82
DAN-style	87.16 ± 5.26	85.99 ± 5.35	0.93	0.92
DAN + cosine	84.12 ± 4.83	82.06 ± 4.83	0.27	0.77
CDAN	42.69 ± 21.17	35.61 ± 21.26	8.56	33.07
MCC	83.86 ± 6.47	79.55 ± 7.52	0.74	0.80
Fixed-schedule Studentized MK-MMD	88.75 ± 5.27	88.18 ± 5.81	0.29	1.28
**Adaptive Studentized MK-MMD (proposed)**	**89.32** ± 5.33	**88.89** ± 5.47	0.94	1.77

**Table 6:** Final-epoch performance and temporal measures on PHM2009. Accuracy and macro-F1 are reported as the mean ± task-averaged sample SD across five seeds (mean in %, SD in percentage points). Last-10 SD and peak–final gap describe within-run variation in percentage points. Bold mean values indicate the highest mean accuracy and macro-F1 in the table.

Method	Accuracy (Mean ± SD)	Macro-F1 (Mean ± SD)	Last-10 SD (pp)	Peak–Final Gap (pp)
Source-only	57.56 ± 2.59	53.92 ± 2.32	0.79	2.40
DAN-style	71.47 ± 2.94	71.65 ± 2.70	0.60	1.35
DAN + cosine	71.17 ± 2.78	71.14 ± 2.58	0.24	1.05
CDAN	56.23 ± 6.70	54.62 ± 6.60	5.14	13.85
MCC	70.26 ± 3.82	70.09 ± 3.76	1.02	2.71
Fixed-schedule Studentized MK-MMD	73.49 ± 3.18	73.71 ± 2.91	0.33	1.23
**Adaptive Studentized MK-MMD (proposed)**	**73.86** ± 3.28	**74.12** ± 3.00	0.59	1.17

**Table 7:** Component and schedule comparisons on PU. Accuracy is reported as the final-epoch mean ± task-averaged sample SD across five seeds (mean in %, SD in percentage points). Last-10 SD describes within-run temporal variation in percentage points. Bold mean accuracy marks the highest value in this table. “No scheduled cooling” changes the learning-rate and alignment-weight schedules together.

Configuration	Accuracy (Mean ± SD)	Last-10 SD (pp)
Fixed-schedule Studentized MK-MMD	88.75 ± 5.27	0.29
Without DSBN	35.96 ± 8.18	0.49
Without Studentization	78.19 ± 5.00	0.30
Without slow statistics	88.69 ± 5.32	0.29
No scheduled cooling	**89.65** ± 5.44	1.55
Adaptive Studentized MK-MMD (proposed)	89.32 ± 5.33	0.94

**Table 8:** Component and schedule comparisons on PHM2009. Accuracy is reported as the final-epoch mean ± task-averaged sample SD across five seeds (mean in %, SD in percentage points). Last-10 SD describes within-run temporal variation in percentage points. Bold mean accuracy marks the highest value in this table. “No scheduled cooling” changes the learning-rate and alignment-weight schedules together.

Configuration	Accuracy (Mean ± SD)	Last-10 SD (pp)
Fixed-schedule Studentized MK-MMD	73.49 ± 3.18	0.33
Without DSBN	69.39 ± 3.43	0.39
Without Studentization	69.92 ± 2.69	0.25
Without slow statistics	73.58 ± 3.16	0.32
No scheduled cooling	72.68 ± 3.90	1.86
Adaptive Studentized MK-MMD (proposed)	**73.86** ± 3.28	0.59

**Table 9:** Matched schedule ablation on transfers 0→1 and 0→2 in each dataset (three seeds per transfer). Accuracy is the mean final-epoch accuracy in percent; Last-10 SD is the mean within-run standard deviation over the final ten epochs in percentage points.

Schedule	PU Accuracy	PU Last-10 SD	PHM Accuracy	PHM Last-10 SD
Matched no cooling	91.07	0.91	78.65	1.35
Joint cooling	91.30	0.98	79.61	0.53
Learning-rate-only cooling	91.53	1.02	79.57	0.56
Alignment-weight-only cooling	91.23	1.02	79.49	1.01

**Table 10:** Sensitivity of the adaptive schedule to the plateau threshold δ and patience *P* on transfers 0→1 and 0→2 in each dataset (three seeds per transfer). Accuracy is reported in percent and last-10-epoch SD in percentage points; cooling epochs are averaged across the six runs.

(δ,P)	PU Acc.	PU SD	PU Cool.	PHM Acc.	PHM SD	PHM Cool.
(0.10,3)	91.07	0.91	0.0	79.65	0.79	13.7
(0.15,3)	91.30	0.98	2.8	79.61	0.53	17.0
(0.20,3)	90.92	0.78	11.0	79.65	0.45	17.8
(0.15,2)	90.84	0.90	3.7	79.61	0.45	18.0
(0.15,4)	91.38	0.93	1.2	79.41	0.57	16.0

**Table 11:** Warm-up sensitivity on transfers 0→1 and 0→2 in each dataset (three seeds per transfer). Values are means across six runs; target accuracy is reported in percent, and alignment loss is the epoch-mean Studentized MK-MMD objective at epoch 50.

Warm-Up (Epochs)	PU Accuracy	PU Alignment Loss	PHM Accuracy	PHM Alignment Loss
0	87.61	3.58	76.32	3.84
10	89.61	3.24	78.21	3.45
20	91.30	3.15	79.61	3.43

**Table 12:** Effect of available record duration on PU transfers 0→1 and 0→2 (three seeds per transfer). Window counts are ranges across the six runs; accuracy and macro-F1 are means in percent. Test-window indices are identical between the two availability settings.

Available Portion	Source Training Windows	Target Adaptation Windows	Accuracy	Macro-F1
Full record	863	863–872	91.30	90.90
First half	431–434	420–431	87.46	87.02

## Data Availability

The data and code used in this study are available from the corresponding author upon reasonable request.
